# Supplementary material for: Content-rich biological network constructed by mining PubMed abstracts
Source: BMC Bioinformatics. 2004 Oct 8;5:147. doi: 10.1186/1471-2105-5-147 (PMC528731; doi:10.1186/1471-2105-5-147)
Supplement: Additional File 2 — The original results of the above study (non-essential files are deleted to keep the file size under the limit set by BMC bioinformatics). [file 1471-2105-5-147-S2.bz2 › chilibotAdditionalFile2/dip50/365ID8663044E6038/html/FGFR2_FGF7.html]

 


 **FGFR2** and **FGF7** 
  
Found 200 abstracts in PubMed, retrieved 50.  
 

 What does Google say? 
 PDF only 
| .edu only 

---

**Interactive relationship** (e.g. stimulation, inhibition, etc)

**Stimulatory relationship**- RESULTS Although KGF  [ **FGF7** ]  is known to target only epithelial cells, all of the three cell lines transduced with K sam  [ **FGFR2** ]  proliferated due to KGF  [ **FGF7** ]  stimulation.  Ref: 11937263 Exp Hematol, 2002
- keratinocyte growth factor  [ **FGF7** ]  quantitative multiplex PCR revealed stimulated keratinocyte growth factor  [ **FGF7** ]  receptor  [ **FGFR2** ]  and androgen receptor messenger RNA expression.  Ref: 11458116 J Urol, 2001
- Both are mainly synthesized by mesenchymal cells and stimulate epithelial cells via KGF  [ **FGF7** ]  receptor KGFR  [ **FGFR2** ]  which is a splice variant of FGFR 2  [ **FGFR2** ] .  Ref: 12565793 Exp Mol Pathol, 2002
- KGF  [ **FGF7** ]  treatment significantly increased production of CFCs derived from K sam  [ **FGFR2** ]  transduced bone marrow cells without causing the exhaustion of immature CFCs.  Ref: 11937263 Exp Hematol, 2002
- Keratinocyte growth factor  [ **FGF7** ]  stimulates the growth and activity of epithelial cells via the keratinocyte growth factor  [ **FGF7** ]  receptor  [ **FGFR2** ] .  Ref: 12351666 J Dent Res, 2002
**Neutral relationship**- **FGF7**  and FGF10 activate only the b isoform of  **FGFR2**  FGFR2b.  Ref: 12591959 Proc Natl Acad Sci U S A, 2003
- A lymphoid cell line BaF KGFR  [ **FGFR2** ]  containing the FGF 7  [ **FGF7** ]  receptor  **FGFR2**  IIIb was treated with FGF 7  [ **FGF7** ]  and with various glycosaminoglycans.  Ref: 12215437 J Biol Chem, 2002
- KGF  [ **FGF7** ]  upregulated EC KGFR  [ **FGFR2** ]  mRNA expression, predominately in the crypt and lower portion of the villus.  Ref: 12757114 JPEN J Parenter Enteral Nutr< MedlineTA>JPEN J Parenter Enteral Nutr, 2002
- Rat kidney organ culture experiments in the presence of a soluble  **FGFR2**  IIIb chimera or a neutralizing antibody to  **FGF7**  suggested an important contribution of FGFs other than  **FGF7**  to the branching program.  Ref: 11731227 Mech Dev, 2001
- These findings suggest that the keratinocyte growth factor  [ **FGF7** ]  receptor  [ **FGFR2** ]  pathway of mesenchymal epithelial interaction could play an important part in the molecular pathogenesis of gingival hyperplasia.  Ref: 12351666 J Dent Res, 2002
- Keratinocyte growth factor  [ **FGF7** ]  receptor  [ **FGFR2** ]  KGFR  [ **FGFR2** ] , also known as fibroblast growth factor receptor FGFR 2  [ **FGFR2** ]  IIIb, is located in many types of epithelial cells and is activated by four known ligands FGF 1, FGF 3, FGF 7  [ **FGF7** ]  also known as KGF  [ **FGF7** ]  and FGF 10 that are predominantly synthesized by mesenchymal cells.  Ref: 12608893 Pathol Int, 2003
- Exposure to both UVB 30 150 mJ cm 2 and CUH 200 microM of NIH3T3 KGFR  [ **FGFR2** ]  KGF  [ **FGF7** ]  receptors transfectants caused a rapid tyrosine phosphorylation and activation of KGFR  [ **FGFR2** ]  similar to that induced by KGF  [ **FGF7** ] , and internalization of the activated receptor.  Ref: 12717419 Oncogene, 2003
- FGF 10 actions are dependent on their binding to the iiib form of FGF receptor  [ **FGFR2** ]  2  **FGFR2**  iiib, also known as keratinocyte growth factor  [ **FGF7** ]  receptor  [ **FGFR2** ]  KGFR  [ **FGFR2** ] .  Ref: 11404772 J Nippon Med Sch, 2001
- CONCLUSIONS KGF  [ **FGF7** ]  could efficiently induce proliferation of hematopoietic cells expressing the K sam  [ **FGFR2** ]  gene without obvious induction of differentiation or exhaustion of immature progenitor cells.  Ref: 11937263 Exp Hematol, 2002
- Keratinocyte growth factor  [ **FGF7** ]  regulates proliferation and differentiation of hematopoietic cells expressing the receptor gene K sam  [ **FGFR2** ] .  Ref: 11937263 Exp Hematol, 2002
- The relative mitogenic efficacy of basic FGF, acidic FGF, or KGF  [ **FGF7** ]  was in correlation with their affinities for the KGF  [ **FGF7** ]  receptor KGFR  [ **FGFR2** ] .  Ref: 12761878 J Cell Biochem, 2003
- Taken together our results demonstrate that exposure to UVB and to oxidant stimuli induces a rapid intracellular production of ROS, which in turn are capable of triggering KGFR  [ **FGFR2** ]  activation and internalization, similar to those induced by KGF  [ **FGF7** ] .  Ref: 12717419 Oncogene, 2003
- Fibroblast Growth Factor Receptor 2  [ **FGFR2** ]  isoform IIIb FGF receptor  [ **FGFR2** ]  2 IIIb is a spliced variant of FGF receptor  [ **FGFR2** ]  2 that binds the ligands FGF 1 and FGF 7  [ **FGF7** ]  with high affinity, and is expressed exclusively by epithelial cells.  Ref: 11593393 Oncogene, 2001
- Dermatan sulfate also enabled FGF 7  [ **FGF7** ]  dependent phosphorylation of mitogen activated protein kinase and promoted binding of radiolabeled FGF 7  [ **FGF7** ]  to  **FGFR2**  IIIb.  Ref: 12215437 J Biol Chem, 2002
- It binds to and activates a receptor tyrosine kinase, the KGF  [ **FGF7** ]  receptor KGFR  [ **FGFR2** ] , which is a splicing variant of the fibroblast growth factor receptor 2  [ **FGFR2** ] .  Ref: 12122441 Histochem Cell Biol, 2002
- These findings suggest that FGF 7  [ **FGF7** ]  and FGF 10 contribute to the regeneration and differentiation of acinar cells and angiogenesis in AP through KGFR  [ **FGFR2** ] .  Ref: 12565793 Exp Mol Pathol, 2002
- Three stable cell lines expressing IIIb  **FGFR2**  demonstrated chemotaxis toward KGF  [ **FGF7** ] , but never proliferated, secreted urokinase, or formed tube like structure by KGF  [ **FGF7** ]  treatment.  Ref: 11735116 Biochem Biophys Res Commun, 2001
- Then we demonstrated that these effects of the mesenchyme were mimicked by fibroblast growth factor 7  [ **FGF7** ]   **FGF7** , a specific ligand of FGFR2IIIb, which is a tyrosine kinase receptor of the FGF receptor  [ **FGFR2** ]  family.  Ref: 11891329 Proc Natl Acad Sci U S A, 2002
- FGF1,  **FGF7** , FGF9, FGF10, FGF11, and FGF18 were also expressed in the pancreas at varying time points and levels, as well as FGF receptor  [ **FGFR2** ]  forms FGFR1b, FGFR1c, FGFR2b, FGFR2c, FGFR3b, and FGFR4.  Ref: 12666204 Dev Dyn, 2003
- Heparin and LMWH bind to low affinity sites on KGF  [ **FGF7** ]  and produce non specific inhibition, while KGFR2 beta IIIb Fc, a soluble chimera of an extracellular KGFR  [ **FGFR2** ]  fragment, is a more specific KGF  [ **FGF7** ]  inhibitor.  Ref: 12529961 Anticancer Res< MedlineTA>Anticancer Res, 2003

**Non-interactive relationship** (e.g. studied together, co-existance, homology, etc.)

- FGF 7  [ **FGF7** ]  did not support cell proliferation in the absence of glycosaminoglycan or with addition of heparan sulfate or chondroitin sulfate A C but did stimulate BaF KGFR  [ **FGFR2** ]  division in the presence of dermatan sulfate or highly sulfated low molecular weight fractions of dermatan.  Ref: 12215437 J Biol Chem, 2002
- The alpha5 integrin, keratinocyte growth factor  [ **FGF7** ]  receptor  [ **FGFR2** ]  and cyclin D1 mRNA levels were higher compared to the controls as early as 2 h after ethanol and 30 min after acetone treatment of the cells.  Ref: 12720008 Arch Dermatol Res< MedlineTA>Arch Dermatol Res, 2002
- Regulation of keratinocyte growth factor  [ **FGF7** ]  receptor  [ **FGFR2** ]  and androgen receptor in epithelial cells of the human prostate.  Ref: 11458116 J Urol, 2001
- Changes in the expression of KGF  [ **FGF7** ]  and KGFR  [ **FGFR2** ]  are less pronounced.  Ref: 11737224 Eur J Clin Invest, 2001
- RESULTS De novo AR expression by PC3 cells restores  **FGFR2**  IIIb isoform expression and sensitivity to  **FGF7**  and FGF2.  Ref: 12430142 Prostate, 2002
- PCR showed keratinocyte growth factor  [ **FGF7** ]  receptor  [ **FGFR2** ]  and androgen receptor expression in epithelial cultured cells but no keratinocyte growth factor  [ **FGF7** ]  expression.  Ref: 11458116 J Urol, 2001
- Proliferation by KGF  [ **FGF7** ]  of murine primary bone marrow cells transduced with K sam  [ **FGFR2** ]  then was examined in liquid culture.  Ref: 11937263 Exp Hematol, 2002
- FGF2,  **FGF7** , and  **FGFR2**  were expressed in neuronal tissue of NG segments as well as in hypertrophic nerves of AG segments.  Ref: 11409166 Pediatr Surg Int, 2001
- Androgen receptor, keratinocyte growth factor  [ **FGF7** ]  receptor  [ **FGFR2** ]  and keratinocyte growth factor  [ **FGF7** ]  messenger RNA expression was measured by polymerase chain reaction PCR.  Ref: 11458116 J Urol, 2001
- Expression of  **FGFR2**  is restricted to domains of advanced osseous differentiation in both Apert syndrome and Pfeiffer syndrome affected cranial skeletogenesis in the presence of fibroblast growth factor FGF 2, but not in the presence of FGF4 or  **FGF7** .  Ref: 11596961 J Neurosurg, 2001
- P63a mutations lead to aberrant splicing of keratinocyte growth factor  [ **FGF7** ]  receptor  [ **FGFR2** ]  in the Hay Wells Syndrome.  Ref: 12692135 J Biol Chem< MedlineTA>J Biol Chem, 2001
- Morphologic observation showed that KGF  [ **FGF7** ]  induced proliferation but did not cause significant differentiation of 32D K sam  [ **FGFR2** ]  cells.  Ref: 11937263 Exp Hematol, 2002
- Treatment with 5 OH 2 D 3 increased the levels of KGFR  [ **FGFR2** ]  in the presence but not in the absence of KGF  [ **FGF7** ] , probably due to inhibition of ligand induced receptor degradation.  Ref: 12761878 J Cell Biochem, 2003
- Our results indicate the possible involvement of both KGF  [ **FGF7** ]  and KGFR  [ **FGFR2** ]  in enhanced epithelial cell proliferative activity and recurrence of cholesteatoma.  Ref: 12533693 Lab Invest, 2003
- MATERIALS AND METHODS The K sam  [ **FGFR2** ]  gene encoding the receptor for keratinocyte growth factor  [ **FGF7** ]  KGF  [ **FGF7** ]  was transduced to three factor dependent hematopoietic cell lines Ba F 2Dcl3, and UT 7 GM using retroviral vector, and their proliferation, differentiation, and intracellular signaling were studied.  Ref: 11937263 Exp Hematol, 2002
- METHODS To quantify the expression of fibroblast growth factor receptor isoforms  **FGFR2**  IIIb,  **FGFR2**  IIIc, FGFR1 IIIc, and fibroblast growth factors FGF2 and  **FGF7**  in normal and tumoral human prostate tissues, and human prostatic epithelial cell lines, we used quantitative real time polymerase chain reaction.  Ref: 12111699 Prostate, 2002
- KGF  [ **FGF7** ]  and KGFR  [ **FGFR2** ]  expression was examined by immunohistochemistry using rabbit anti human KGF  [ **FGF7** ]  and anti human KGFR  [ **FGFR2** ]  polyclonal antisera raised in our laboratories against synthetic peptides corresponding to parts of human KGF  [ **FGF7** ]  and KGFR  [ **FGFR2** ] , respectively.  Ref: 12533693 Lab Invest, 2003
- ... neither one stimulated FGF 1 signaling via FGFR1 or FGF 7  [ **FGF7** ]  via  **FGFR2**  IIIb.  Ref: 11714710 J Biol Chem, 2002
- Parallel western blot analysis revealed that KGFRs were tyrosine phosphorylated both at early and late steps of internalization, suggesting that KGF  [ **FGF7** ]  and KGFR  [ **FGFR2** ]  remain associated in active complexes through the endocytic pathway.  Ref: 12122441 Histochem Cell Biol, 2002
- Epithelial KGF  [ **FGF7** ]  receptor KGFR  [ **FGFR2** ]  mRNA expression was studied using real time reverse transcriptase polymerase chain reaction RT PCR.  Ref: 12757114 JPEN J Parenter Enteral Nutr< MedlineTA>JPEN J Parenter Enteral Nutr, 2002
- The difference in keratinocyte growth factor  [ **FGF7** ]  receptor  [ **FGFR2** ]  and androgen receptor gene expression after treatment with and without keratinocyte growth factor  [ **FGF7** ]  and flutamide were determined by quantitative multiplex PCR and quantitated using densitometry analysis.  Ref: 11458116 J Urol, 2001
- In vivo inhibition of keratinocyte growth factor  [ **FGF7** ]  receptor  [ **FGFR2** ]  expression by estrogen and antagonism by progesterone in the mouse mammary gland.  Ref: 11691652 J Endocrinol, 2001
- Examination of hormonal regulation of the fibroblast growth factor FGF 7  [ **FGF7** ]   **FGFR2**  IIIb receptor system in the mammary gland reveals that mammogenic hormones differentially regulate the synthesis of stromal growth factors and their epithelial receptors.  Ref: 11897505 J Steroid Biochem Mol Biol, 2002
- Our results support the notion that the paracrine KGF  [ **FGF7** ]  KGFR  [ **FGFR2** ]  system in the skin can act in concert with the autocrine vitamin D system in keratinocytes to promote keratinocyte proliferation and survival under situations of stress and injury.  Ref: 12761878 J Cell Biochem, 2003
- Tumour cells expressed  **FGF7**  mRNA and protein, and low levels of  **FGFR2**  mRNA.  Ref: 12569015 Int J Radiat Biol, 2003
- UVB induced activation and internalization of keratinocyte growth factor  [ **FGF7** ]  receptor  [ **FGFR2** ] .  Ref: 12717419 Oncogene, 2003
- Further, we observed that the level of KGF  [ **FGF7** ]  receptor KGFR  [ **FGFR2** ]  expression in ER positive cells was much greater than in the ER negative cell lines.  Ref: 11688962 Clin Exp Metastasis, 2000
- In contrast, only relatively long oligosaccharides supported receptor binding and activation in the FGF 1.FGFR1 or FGF 7  [ **FGF7** ] . **FGFR2**  IIIb setting.  Ref: 11714710 J Biol Chem, 2002
- Murine brain capillary endothelial cells, denoted IBE cells, express small amounts of IIIc  **FGFR2** , which is not activated by keratinocyte growth factor  [ **FGF7** ]  KGF  [ **FGF7** ] .  Ref: 11735116 Biochem Biophys Res Commun, 2001
- KGF  [ **FGF7** ]  and KGFR  [ **FGFR2** ]  mRNA expression were increased after 14 28 days 5 fold and then returned to control levels.  Ref: 11737224 Eur J Clin Invest, 2001
- These peptide growth factors and their receptors c met and keratinocyte growth factor  [ **FGF7** ]  receptor  [ **FGFR2** ]  KGFR  [ **FGFR2** ]  are overexpressed in pancreatic cancer.  Ref: 11737224 Eur J Clin Invest, 2001
- Previously, FGF 7  [ **FGF7** ]  mRNA expression was reported in VSMC, but KGFR  [ **FGFR2** ]  mRNA was not detected.  Ref: 12608893 Pathol Int, 2003
- The effects of ethanol and acetone on the mRNA levels of genes characteristic for proliferating keratinocytes such as alpha5 integrin, keratinocyte growth factor  [ **FGF7** ]  receptor  [ **FGFR2** ]  and cyclin D1 were studied by reverse transcriptase polymerase chain reaction.  Ref: 12720008 Arch Dermatol Res< MedlineTA>Arch Dermatol Res, 2003
- In the present study, we investigated the possible involvement of KGF  [ **FGF7** ]  and its receptor, KGFR  [ **FGFR2** ] , in the pathogenesis of cholesteatoma using in situ hybridization and immunohistochemistry, respectively.  Ref: 12533693 Lab Invest, 2003
- The endocytic pathway followed by the keratinocyte growth factor  [ **FGF7** ]  receptor  [ **FGFR2** ] .  Ref: 12122441 Histochem Cell Biol, 2002
- Keratinocyte growth factor  [ **FGF7** ]  receptor  [ **FGFR2** ]  is up regulated in cyclosporin A induced gingival hyperplasia.  Ref: 12351666 J Dent Res, 2002
- To test this hypothesis, TGFbeta1, TGFbeta2 TGFbeta3, TGFbetaR I, TGFbetaR II, TGFalpha, EGF, EGFR, KGF  [ **FGF7** ]  and KGFR  [ **FGFR2** ]  gene and protein expression were analyzed in dorsal and ventral lobes of aging rat prostates,, 1 4, and 28 30 months.  Ref: 11906188 Biochem Biophys Res Commun, 2002
- FGF2 and  **FGF7**  for FGFs, FGFR1 and  **FGFR2**  for FGFRs, NCAM, L1CAM, and N cadherin for CAMs.  Ref: 11409166 Pediatr Surg Int, 2001
- The present study was therefore carried out to determine whether expression of the keratinocyte growth factor  [ **FGF7** ]  receptor  [ **FGFR2** ]  is up regulated in gingival hyperplasia compared with normal gingiva.  Ref: 12351666 J Dent Res, 2002
- We found reductions of Clara cell specific protein and keratinocyte growth factor  [ **FGF7** ]  receptor  [ **FGFR2** ]  mRNAs in both terminal bronchiolar epithelium and whole lung homogenates 7 d after bleomycin.  Ref: 11588004 Am J Respir Cell Mol Biol, 2001
- MATERIALS AND METHODS In the present study three KGF  [ **FGF7** ]  inhibitors were employed Heparin, Innohep, a low molecular weight heparin LMWH and KGFR2 beta IIIb Fc, a chimeric KGFR  [ **FGFR2** ]  fragment.  Ref: 12529961 Anticancer Res< MedlineTA>Anticancer Res, 2001
- **Fgf7**  and Fgf10, the main ligands for  **FgfR2**  IIIb, are expressed in the mesenchyme surrounding the thymic epithelial primordium, and Fgf10 deficient mice also exhibit impaired thymic growth.  Ref: 11489975 J Immunol, 2001
- Assessment of the proliferative activity of cholesteatoma using the labeling index for Ki 67 showed a significantly higher Ki 67 labeling index 66% in KGF  [ **FGF7** ]  KGFR  [ **FGFR2** ]  cases than other cases.  Ref: 12533693 Lab Invest, 2003
- The expression of iNOS, KGF  [ **FGF7** ]  and KGFR  [ **FGFR2** ]  in laser wounds was more intense than in electrocautery wounds.  Ref: 11858949 J Dermatol Sci, 2002
- Therefore, the increase in the number of dividing cells and in the expression level of iNOS, KGF  [ **FGF7** ]  and KGFR  [ **FGFR2** ]  may induce earlier and thicker reepithelization in laser wounds than in electrocautery and scalpel wounds.  Ref: 11858949 J Dermatol Sci, 2002
- Expression of keratinocyte growth factor  [ **FGF7** ]  receptor  [ **FGFR2** ]  KGFR  [ **FGFR2** ]   **FGFR2**  IIIb in vascular smooth muscle cells.  Ref: 12608893 Pathol Int, 2003
- In epithelial tissues, keratinocyte growth factor  [ **FGF7** ]  KGF  [ **FGF7** ]  or FGF 7  [ **FGF7** ]  originates in the stroma while its receptor KGFR  [ **FGFR2** ]  or  **FGFR2**  IIIb is present only in the epithelium.  Ref: 11691652 J Endocrinol, 2001
- There was a significant correlation between KGF  [ **FGF7** ]  KGFR  [ **FGFR2** ]  expression and recurrence.  Ref: 12533693 Lab Invest, 2003
- We have reported that normal human salivary gland derived epithelial cells exclusively express keratinocyte growth factor  [ **FGF7** ]  receptor  [ **FGFR2** ]  KGFR  [ **FGFR2** ] .  Ref: 11562460 Proc Natl Acad Sci U S A, 2001
- The gene expression of HGF and keratinocyte growth factor  [ **FGF7** ]  KGF  [ **FGF7** ]  in gingival connective tissue, and c met, HGFA and KGF  [ **FGF7** ]  receptor KGFR  [ **FGFR2** ]  in gingival epithelial tissues was monitored using RT PCR.  Ref: 12613501 J Oral Sci, 2002
- Vitamin D enhances mitogenesis mediated by keratinocyte growth factor  [ **FGF7** ]  receptor  [ **FGFR2** ]  in keratinocytes.  Ref: 12761878 J Cell Biochem, 2003
- A role for the perlecan protein core in the activation of the keratinocyte growth factor  [ **FGF7** ]  receptor  [ **FGFR2** ] .  Ref: 11563979 Biochem J, 2001
- Growth inhibition by keratinocyte growth factor  [ **FGF7** ]  receptor  [ **FGFR2** ]  of human salivary adenocarcinoma cells through induction of differentiation and apoptosis.  Ref: 11562460 Proc Natl Acad Sci U S A, 2001
- Thus, KGF  [ **FGF7** ] , KGFR  [ **FGFR2** ]  and or the KGF  [ **FGF7** ]  signaling pathway may be important therapeutic targets for the treatment or prevention of breast cancer metastasis.  Ref: 11688962 Clin Exp Metastasis, 2000
- Wounds were examined by transmission electron microscopy, the detection of free 3 OH DNA ends and immunohistochemistry of proliferating cell nuclear antigen PCNA, inducible nitric oxide synthase iNOS, keratinocyte growth factor  [ **FGF7** ]  KGF  [ **FGF7** ]  and keratinocyte growth factor  [ **FGF7** ]  receptor  [ **FGFR2** ]  KGFR  [ **FGFR2** ] .  Ref: 11858949 J Dermatol Sci, 2002
- The low  **FGFR2**  expression as well as the  **FGF7**  expression in the tumour cells may contribute to their resistance to rHuKGF treatment.  Ref: 12569015 Int J Radiat Biol, 2003
- The objective of this study was to assess the effect of repifermin on in vitro tumor cell proliferation and in vivo tumor growth using a variety of human carcinoma cell lines with differing growth rates and levels of KGF  [ **FGF7** ]  receptor KGFR  [ **FGFR2** ]  expression.  Ref: 12203102 Cancer Chemother Pharmacol, 2002
